# Pig Farmers' Perceptions of Economic Incentives to Control *Salmonella* Prevalence at Herd Level

**DOI:** 10.3389/fvets.2021.647697

**Published:** 2021-04-16

**Authors:** Jakob Vesterlund Olsen, Tove Christensen, Jørgen Dejgaard Jensen

**Affiliations:** Department of Food and Resource Economics, University of Copenhagen, Copenhagen, Denmark

**Keywords:** questionnaire, *Salmonella*, economic incentives, zoonosis, pig farmers

## Abstract

This paper investigates how perceived costs and benefits of *Salmonella* control among Danish pig farmers affect the farmers' choice of action toward reducing the prevalence of *Salmonella* in their herds. Based on data from an online questionnaire involving 163 Danish pig farmers, we find a considerable uncertainty among pig farmers about the perceived effects of the *Salmonella* reducing actions. The results indicate large variations in the perceived costs of implementing different types of *Salmonella* reducing actions (management-, hygiene- and feed-related). For some cases, farmers associate net benefits and positive productivity effects with implementation of the actions while studies by the industry indicate net costs to the farmers. Differences among farmers support the idea of an outcome-based *Salmonella* penalty scheme but the large uncertainties about costs and effects of actions toward *Salmonella* control might hamper the effectiveness of such a penalty scheme as a regulatory instrument to affect farmer behavior.

## Introduction

*Salmonella* is a zoonosis causing illness for many people globally. According to EFSA and ECDC (1), almost 88,000 confirmed cases of Salmonellosis in humans were reported in 2019 in the European Union. Poultry meat and eggs are the most prevalent sources of food-borne *Salmonella* contamination but pork is also a significant contributor to Salmonellosis in humans (2). *Salmonella* is typically not associated with clinical disease for pigs who are healthy carriers of the bacteria (3) but outbreaks of Salmonellosis among piglets can be associated with reduced daily weight gain (4). Overall, the prevalence of *Salmonella* bacteria does not *per se* incentivize farmers to reduce *Salmonella* on productivity grounds. Hence, the main costs of *Salmonella* in pigs and in pork seem to be carried by the consumers. As farmers do not have economic incentives to include risk of human infection from contaminated pork in their production decisions, human infections of Salmonellosis from eating pork can be considered an externality effect of pig production (5).

In order to reduce societal costs of human illness due to foodborne *Salmonella*, action plans have been initiated at EU level (6, 7) as well as at national levels (8). Denmark has had action plans for reducing the prevalence of *Salmonella* in pigs and in pork since 1995. The Danish approach to monitoring and controlling *Salmonella* in pork involves interventions in all parts of the food supply chain both at farm level, feed companies and at abattoirs. The goal is to maintain prevalence in carcasses below 1% (8, 9).

In 1998, Denmark introduced an economic penalty scheme where farmers pay a penalty when delivering pigs to the abattoir with high *Salmonella* prevalence (10). Thereby, direct economic incentives were introduced to motivate farmers who deliver pigs to the abattoirs to take action to reduce risks of carcasses containing *Salmonella* bacteria. While several studies suggest that controlling *Salmonella* at the abattoirs is more cost-effective than using farm level actions (9, 11, 12), they also stress the importance of keeping a low to moderate prevalence level at farm level (13, 14). Therefore, even though abattoir-level actions are more cost-effective, there seems to be a consensus to use a combination of pre- and post-harvest actions to reach target levels of *Salmonella* in pork (10, 15). The target level is keeping the prevalence of Salmonella below 1% of carcasses at the abattoir (16).

The *Salmonella* herd surveillance depends on whether it is a breeding herd, a sow herd or a finisher herd. All Danish herds delivering more than 200 finisher pigs to abattoirs per year are assigned a *Salmonella* level. The *Salmonella* level that can take the values one to three, with one being the best level without or with only low prevalence of *Salmonella*. The *Salmonella* level of a herd is determined at the abattoir, where a number of meat juice samples from each herd regularly are tested for antibodies against *Salmonella* using serological analysis (17, 18). If the sample shows that more than 40% of pigs from a herd are tested positive with antibody levels above a given threshold, the herd is placed in level two. If more than 65% of the pigs in a herd are tested positive, the herd is placed in level three. The percentages of positive tests are calculated as a weighted average of the last 3 months, with the latest month having a weight of 0.6, the month before a weight of 0.3 and the month prior to this a weight of 0.1.

The penalties that farmers delivering finisher pigs to the abattoir pay in a given month are determined by the assigned *Salmonella* levels. For pigs delivered from herds in level two, a penalty of 2% of production value is retained at the abattoir. The penalty increases to 4% of the production value being withheld for pigs from herds in level three. If the herd has been in level three for more than 6 months, the penalty increases to 6%. Finally, if slaughter pigs have been subject to a 6% penalty for more than 6 months and still is in level three, an 8% penalty is withheld at the abattoir. The scheme aims to induce pig farmers to think of *Salmonella* control as an economic problem where costs of implementing actions to reduce *Salmonella* risks at least to some extent are weighted against the benefits of having low levels of *Salmonella* in the herds. Here, avoided penalty constitutes the economic benefits of a low level of *Salmonella*. According to economic theory, the penalty scheme has the possibility to induce efficient reductions in *Salmonella* levels as it provides economic incentives for *Salmonella* control while at the same time allowing the individual farmers the freedom to choose the actions that are most cost-effective considering their specific herd characteristics. The penalty scheme provides a direct economic incentive for farmers who deliver finisher pigs to the abattoir to try to avoid *Salmonella*.

All pig herds (not only finisher herds) are placed in one of three *Salmonella* categories. Category A is for herds without *Salmonella*, category C is for herds that have *Salmonella* Typhimorium, Derby, Infantis, or Enterica while category B is for herds with other types of *Salmonella* than those grouped in category B (17, 19).

Sow herds are tested for *Salmonella* if the finisher herds they deliver piglets to are in level two or three. In this case, it is mandatory to conduct bacteriological testing of fecal samples in the sow herd delivering piglets. Thereby, information about *Salmonella* status of a sow herd is available for potential buyers of piglets. This information is intended to provide incentives for pig farmers to buy piglets from category A herds. It is not mandatory to buy piglets from category A herds but the information is available for owners of finisher pig herds if they want to do so.

Breeding herds are categorized as A, B, or C herds, based on serological testing of blood samples from young breeding animals (4–7 months old). If the tests indicate *Salmonella* prevalence above a given threshold, then also bacteriological testing of pen (fecal) samples are carried out (17).

A large number of studies have investigated risk factors for *Salmonella* prevalence in pig herds and actions to reduce *Salmonella* prevalence in pig herds. These include studies from Spain (20), Germany (21), Canada (22), the US (23), and Denmark (24–27). The studies point toward three overall types of actions for controlling *Salmonella* (28). One type of *Salmonella* control concerns the feed and water where for example adding organic acids to water and feed has shown to be protective against *Salmonella* while the use of pelleted feed is perceived to increase *Salmonella* risks (2, 15, 21, 23–27, 29, 30). A second type of *Salmonella* control actions relates to the management procedures where all-in/all-out production systems (31) and only buying *Salmonella* free piglets have been shown to reduce *Salmonella* prevalence in herds (32). The third type of farm-level *Salmonella* controlling actions includes hygiene-related actions such as intensive cleaning and disinfection of pens between batches and having a high level of rodent control (33, 34).

Alas, research findings are not always transformed into practice. One of the reasons could be lack of information flow from researchers to farmers. While there seems to be agreement among experts in Denmark (18) that many of the suggested actions can reduce *Salmonella* prevalence in pig herds, and also agreement among experts about which actions are the most effective, there is limited general advice of the effectiveness of the individual actions in practice on the individual farms. Other reasons for differences between research findings and practice among pig farmers regarding *Salmonella* control actions could include differences in farm specific costs of implementing *Salmonella* control actions (actual as well as perceived costs), additional resource costs for farmers in changing practices, mistrust in the perceived effectiveness of suggested control actions or lack of awareness of the problem (35). These potential reasons for not implementing available *Salmonella* controlling actions pinpoint the importance of involving social science in biosecurity research.

A few social science studies involving pig farmers' perceptions and self-reported behavior regarding biosecurity were found. Alarcon et al. (35) interviewed 20 British pig farmers and found that lack of awareness and knowledge regarding research scientific outputs being barriers for efficient control. Marier et al. (36) found in a British study involving four pig farmers, that the farmers did feel a responsibility for producing *Salmonella* free pigs but lacked confidence in the proposed control actions being effective. A Danish survey involving 138 pig and dairy farmers found that the farmers' were mainly motivated to improve their biosecurity by a desire to reduce the risk of having sick animals and to improve their economic performance and the welfare of their animals. Fewer in the sample of farmers mentioned legislation as a reason for improving biosecurity. The sampled farmers pointed toward a need for more practical solutions on how to prevent disease outbreaks in their herds (37).

We know that a number of actions for controlling *Salmonella* in herds are presently used and have been used by Danish farmers. However, as it is voluntary to choose which actions to implement it is not known how widespread the use of the individual actions are, why some farmers choose specific actions, and what rationales these choices are based upon. In particular, little is known about how economic incentives affect farmers to maintain a low level of *Salmonella* in their herds.

The overall purpose of the paper is to improve our understanding of the extent to which the economic incentives in Danish *Salmonella* action plan induce pig farmers to implement actions aiming to reduce the *Salmonella* prevalence in their herds. To address this overall research purpose, the following research questions (RQ) are answered with reference to Danish pig production:

RQ1 To what extent do existing expert-based estimates of costs and benefits of *Salmonella* control indicate incentives for reducing *Salmonella* prevalence at herd level?

RQ2 How do pig farmers perceive costs and benefits of *Salmonella* control?

RQ3 How are pig farmers' choice of action toward *Salmonella* control affected by attitudes and farm-specific factors?

The research questions were addressed using a combination of surveillance data and an online survey. The survey involved 163 Danish pig farmers with an over representation of farmers having experienced high *Salmonella* prevalence (more details about selection criteria are provided in the “Material and methods” section). Our contribution is to improve the understanding of pig farmers' perceptions and behavior toward *Salmonella* control actions at farm level using an economic framework of costs and benefits of *Salmonella* control. We view costs and benefits broader than direct changes in income and expenditures in that we include costs related to reluctance of changing habits, efforts involved in information acquisition and time resources as potential costs of *Salmonella* control. Moreover, we investigate to what extent differences in attitudes and herd specific characteristics can explain differences in choice of *Salmonella* control actions. We have studied Danish pig farmers as a case, with particular focus on investigating the incentives to control *Salmonella* induced by the Danish penalty scheme.

## Overview of *Salmonella* Prevalence in Danish Pig Herds

An overview of the *Salmonella* levels of Danish pig herds between 2011 and 2018 is presented in Table 1 using data from the Danish Central Husbandry Register (CHR) and the affiliated Danish Zoonosis Register. The CHR register holds information about where pigs are, how many pigs there are on each site, movement of pigs as well as registrations of veterinary events (38). The Danish Zoonosis Register holds information about the *Salmonella* status on a monthly basis for all pig herds with more than 200 pigs slaughtered per year in Denmark (39). In 2018, 85% of the farms remained at the lowest prevalence level throughout the year (level 1). Looking across all years and only including herds that have been in the dataset for at least 5 years, 43% of the herds stayed in level one throughout all years, 29% were at some point in time in level two but not in level three and 28% had been in level three.

**Table 1:** Distribution of Danish pig herds according to their *Salmonella* levels based on highest level during a year.

**Description**	**No. of herds**	***Salmonella*** **status [percent of herds]**		
		**Level 1**	**Level 2**	**Level 3**
Highest *Salmonella* level in 2018	8,459	85%	11%	4%
Highest *Salmonella* level (2011–2018)	5,074	43%	29%	28%

*Level 1: sero-prevalence <40, level 2 sero-prevalence between 40 and 65, level 3: sero-prevalence >65. Data: 2011–2018. Only the 5,074 herds that have been in the dataset for at least 5 years from 2011 through 2018 are included in the latter calculation. Own calculations based on data from the Danish Zoonosis Register*.

With only 4% being in *Salmonella* level three in 2018 but 28% having been in level three at some point during a period of at least 5 years indicates that it is not the same farms which are constantly in level three. This observation is supported by statistics in Table 2, which shows that herds are around 2 months (on average) in *Salmonella* level two or three before they return to a lower level. The relatively short period of time in which herds have a higher *Salmonella* level could reflect that *Salmonella* in a herd dies out without reference to *Salmonella* control initiatives thereby contributing to improved *Salmonella* status. It could also reflect that farmers successfully have implemented actions attempting to reduce the *Salmonella* prevalence in their herds incentivized, possibly by the penalty scheme, to do so. The relatively large percentage of herds that have been in *Salmonella* levels two or three over the years could also point toward a great deal of randomness surrounding the *Salmonella* prevalence in the herds.

**Table 2:** Estimated period that a Danish pig herd has a high *Salmonella* level.

**Shift of level**	**Days**	**Herds**
From 3 to 2	55.3	955
From 3 to 1	70.3	1,054
From 2 to 1	65.0	3,049

*Own calculations based on data from the Danish Zoonosis Register. Data from 2011 to 2018. Only the 5,074 herds that have been in the dataset for at least 5 years from 2011 through 2018 are included in the calculation*.

## Theoretical Framework

The empirical estimations of costs and benefits of *Salmonella* control at farm level and the inclusion of attitudes and perceptions were guided by a theoretical economic model. The model describes pig farmers' choice of *Salmonella* control actions in a stochastic setting. We take as a starting point that farmers seek to maximize their expected profit per finisher pig. A partial comparative static model is used where the only choice variable is the level of *Salmonella* control *a*, which can be influenced through a set of actions, *x*. We assume that *Salmonella* prevalence *s(a)* is a decreasing function of *Salmonella* control *a*. Uncertainty about the effect of *Salmonella* control is captured by assuming that the *Salmonella* prevalence is a stochastic function of control level *a*, which in turn is a function of actions *x*, with the cumulative distribution function *F* as shown in Equation (1):

(1) $$\begin{array}{l} P\left(s\leq S|a\left(x\right)\right)=F\left(S|a\left(x\right)\right) \end{array}$$

Costs associated with *Salmonella* controlling actions are captured by a function *c(x)*. Costs due to changed feeding or management might involve costs in terms of reduced feed conversion rates, increased use of labor and/or antibiotics. Potential changes in production output due to changes in *Salmonella* prevalence were also incorporated although in the case of Danish pig production it is usually not assumed that the *Salmonella* prevalence has an effect on output level (11). Production output, which is the number of pigs for slaughter, is a function of *Salmonella* control action *y(x)*.

The *Salmonella* penalty scheme is included in the theoretical framework as a reduction in payments if *Salmonella* prevalence exceeds a certain threshold mimicking a shift from level one to level two (or from level two to level three). If the *Salmonella* prevalence is above a threshold $\bar{S}$ (i.e., $s\geq \bar{S}$), a penalty in the form of the price reduction δ per pig is levied on the producer. The price per pig is denoted *p*. Given these assumptions, the producer's expected profit function per finisher pig can be written as Equation (2):

(2) $$\begin{array}{l} E\left[\pi \left(a\right)\right]=\left(F\left(\bar{S}|a\left(x\right)\right)\cdot p+\left(1-F\left(\bar{S}|a\left(x\right)\right)\right)\cdot \left(p-\delta \right)\right)\\ \;\cdot y\left(x\right)-c\left(x\right) \end{array}$$

The first-order derivative of this profit function with respect to the intervention action *x*_*j*_

(3) $$\begin{array}{l} \frac{\partial E\left[\pi \right]}{\partial x_{j}}=\frac{\partial F}{\partial a}\frac{\partial a}{\partial x_{j}}\cdot \delta \cdot y\left(x\right)+\left(\left(p-\delta \right)+F\left(\bar{S}|a\left(x\right)\right)\cdot \delta \right)\cdot \\ \;\frac{\partial y}{\partial a}\frac{\partial a}{\partial x_{j}}-\frac{\partial c}{\partial x_{j}} \end{array}$$

The derivative with respect to action *x*_*j*_ represents the net increase in expected profit due to the action. If the derivative is positive, it will be expected to be profitable for the farmer to undertake action *x*_*j*_, whereas it is not expected to be profitable if the derivative is negative. This derivative represents the change in expected profit due to a change in the action variable and has three main components:

- Effect on the expected sales price due to changed probability of facing price penalty δ, which is affected by the impact of *x*_*j*_ on the *Salmonella* prevalence distribution.
- Effect on the output (number of finisher pigs).
- Effect on control costs per finisher pig.

This theoretical framework captures some of the complexities in a real decision process in that the decision to undertake action *x*_*j*_ reflects the farmer's trade-off between these three components. Additionally, the model captures that there may be uncertainty about all of the variables and that control decisions will often to some extent rely on the farmer's subjective perception of these components.

If the functional forms of the distribution function $F\left(\bar{S}|a\right)$, and of the *Salmonella* control *a*(*x*) were specified, Equation (3) could be rearranged and *x*_*j*_ could be identified as a function *f*_*j*_ :

(4) $$x_{j}=\{\begin{array}{c} f_{j}\left(p,\delta ,\frac{\partial y}{\partial x_{j}},\frac{\partial c}{\partial x_{j}}\right),\;if\;\frac{\partial E\left[\pi \right]}{\partial x_{j}}>0\;\\ 0\;otherwise \end{array}$$

To keep the concepts relatively simple and focus on the trade-offs between different effects of *Salmonella* control, we have formulated the theoretical model in a static version where development over time is not included. It may well be imagined that farmers use their experience in one period to improve their understanding of these effects over time. For example, if a price penalty encourages the farmer to undertake an action in one period, the farmer may gain insights in this action's impacts on output and costs which may then lead to updated perceptions of these effects in subsequent periods. Thereby, an action initially undertaken to avoid the price penalty could also prove to be economically attractive to maintain even after the herd is back in *Salmonella* level 1 again.

We now turn to a description of the empirical analyses. They involve a description of data sources and how we have used them to answer the research questions.

## Materials and Methods

### Data and Method Used to Address RQ1

#### Costs of *Salmonella* Control

Altogether, 12 *Salmonella* controlling actions were included in the analysis based on the literature study (27, 40–43) and interviews with two experts from the pig sector. The actions represent management, hygiene and feed actions. See Table 3.

**Table 3:** Description of the 12 *Salmonella* actions and the types of costs involved.

**Action**	**Description of types of costs**
Buy pigs from herds with low *Salmonella* level	More expensive piglets. Some pig farmers include in their contracts that piglet sellers pay the penalty if the finishers are subject to penalty at the abattoir (44). Costs not estimated.
All in-all out/systematic shifting of batches	Only some stables are suitable for all in-all out shifts. It requires that finisher stable is divided into sections. We assumed that all new or renovated stables use this action as it has productivity gains (42). Costs are not estimated as it will not be implemented in stables that are not build for it already and for new buildings, the action will be implemented for productivity reasons.
Extra good hygiene when new batches are introduced	Additional labor costs. We have assumed that a herd with 200 finishers at a time in each section use 2 h additional cleaning between batches with hourly rate at 25.6 Euro/h (45). Additional expenses to material electricity and a high-pressure cleaner are estimated to 13.4 Euro/batch or 34 Eurocents/finisher.
Feed with organic acid	Direct expenses for adding organic acid to the feed for a finisher are estimated to 1.23 Euro per finisher.
Using fermented dry feed	Costs not estimated due to lack of data.
Using fermented wet feed	Costs of using fermented wet feed by using a fermentation tank. Reduced (better) feed conversion ratio is expected but still net costs of 40 cents per finisher due to investments in fermentation tank etc. (43)
Rough milled feed	Costs depend on how roughly milled the feed is and whether home-mixed or readymade feed is used. The costs are mainly related to increased feed conversion ratio. We have estimated the costs to 1.21 Euro per finisher based on results from Jørgensen et al. (26) and Sloth et al. (40).
Feed with high barley content	Costs due to increased feed conversion ratio. Estimated costs of 94 cents per finisher pig based on Jørgensen et al. (27).
Home mixed feed	Costs of using home mixed feed depend highly on whether the farmers has the facilities to do so. Hence, the costs are difficult to convert to variable costs per finisher. Costs are not estimated.
Acidified drinking water	Direct expenses for buying acids that is added to the drinking water. In some cases also capital costs are needed for investing in a mixer. Additional capital costs might be needed if the pipes must be changed to a non-corrosive material. If pipes are not changed, then we estimate that costs for a finisher are 1.32 Euro/finisher. Otherwise, costs are higher.
High hygiene for workers, visitors, dogs, cats, tools	Primarily, labor costs. For an average farm with an extra use of labor of 10 min per day this is estimated to be 28 cents/finisher.
Rodent control	Subscription costs for private rodent control company to supervise and eradicate rodents on the farm. Costs depend on farm size. Costs estimated to 13 Eurocents/finisher for an average farm.

*Own calculations based on literature*.

The cost analyses were carried out as partial analyses with the implicit assumption that costs of implementing multiple *Salmonella* controlling actions are found by adding costs of individual actions. As there might be synergies when implementing multiple actions, this approach is likely to overstate aggregated costs. On the other hand, the only cost data available are based on farm trials where actions have been implemented as it is likely that such farms have lower than average costs. This part of the estimation might understate cost estimates. It should be noted, that not only are the cost estimated uncertain, the effectiveness regarding the effect on *Salmonella* prevalence is also uncertain. Costs of *Salmonella* controlling actions include monetary expenses, estimates of required time allocated to carry out each action, and the effect on productivity. The estimated productivity losses or gains from implementing the individual actions are to a large degree based on the pig sectors' own estimates from the farm trials. As the trials were carried out to guide farmers to choose the most cost-effective action to control *Salmonella*, we do not expect systematic bias in the estimates obtained from experts working in the pig sector. Table 3 presents the actions together with a short description of the types of costs included.

The farms do not have the same opportunities to implement all of the actions in the short run. As an example, farms where the production units are separated into sections have the options to reduce or even eliminate infection between batches. Therefore, an action as “All in-all out/systematic shifting of batches” is only realistically applicable in herds with separated sections. Only costs related to actions, which are applicable to all farmers are estimated. Consequently, costs for four actions were not calculated: All in-all out/systematic shifting of batches, buy piglets from herds with low *Salmonella* level, using fermented wet feed and using home mixed feed. The cost estimates are presented as industry averages.

#### Benefits of *Salmonella* Control

The benefit of *Salmonella* control is the money saved by not having to pay a penalty. Estimating benefits of the individual *Salmonella* controls would require data on the effectiveness of individual actions to reduce Salmonella prevalence. We do not have this kind of data. Instead, benefits of being in *Salmonella* level one as opposed to levels two or three have been estimated. Using this framework, cost estimates based on individual control actions are compared with benefits based on *Salmonella* prevalence in the herd. This approach is obviously not ideal but highlights the imperfect information that often is present when farmers have to make their decisions.

Costs of delivering pigs while being in *Salmonella* level two or three is a percentage of the price of a pig delivered to the abattoir. According to SEGES (46), the price for a standard pig is 134 Euro in 2017. The penalty scheme reduces payments per pig delivered for all pigs delivered in a batch when *Salmonella* prevalence in the herd is above the given limit. Thereby, the benefits depend on the number of pigs delivered to the abattoir, the *Salmonella* status of the herd, and the period of time that the herd has been in level two or three. In order to estimate the penalty costs at farm level for an average farm, we needed an indicator of the yearly production rather than the number of animals at a given point in time on the farm as registered in the CHR, so we used industry statistics to link number of animals to yearly production (46).

### Data and Methods Used to Address RQ2 and RQ3

#### Data Collection

In order to address RQ2 and RQ3, a questionnaire survey was conducted in a sample of Danish pig farmers. Information about herd size, type of herd, and *Salmonella* level was available from the CHR and Zoonosis registers. Using the same categorization as the CHR, we included finisher production, integrated production (producing piglets, weaners and finishers), other production (e.g., piglets only, weaners only, piglets and weaners) and breeding herds. Thereby, we included producers who deliver pigs to the abattoir and consequently can be directly affected by a penalty (integrated and finisher productions) as well as producers who are not directly affected by the risk of a penalty (e.g., piglet producers and breeders). As there are rather few breeders, they are overrepresented in the sample. Furthermore, to make sure to enroll farmers with experience in dealing with *Salmonella*, herds in *Salmonella*-level two or three were also overrepresented in the sample. Altogether, we invited 440 pig herd owners to fill out the questionnaire. The distribution of the 440 herds and the distribution of farmers returning the questionnaire is shown in Table 4. The data were collected between November 16 and December 15 2018 using one re-invitation midways. With 163 herd owners returning the questionnaire, we obtained a response rate of 37%. Unfortunately, we do not have data on those who chose not to respond to the invitation.

**Table 4:** Distribution of Danish pig herds and of herds in the sample categorized according to *Salmonella* level.

	***Salmonella* level 1**	***Salmonella* level 2**	***Salmonella* level 3**	**Total**
Piglet production	811	58	20	889
Finisher pig production	1,856	298	101	2,255
Integrated production	87	23	5	115
Other production	422	72	34	528
Total	3,176	451	160	3,787
Invited in the survey	140	130	130	400
Respondents	52	45	48	145
Breeding herds invited				40
Breeding herds respondents				18
Respondents total				163

*Herds with more than 60 finishers in stock (estimated to deliver more than 200 finishers in a year) were included. A total of 3,787 herds of which 400 were invited in the survey. Also 40 breeding herds were invited. They are listed separately as they do not deliver pigs for slaughter and are not assigned Salmonella levels. Data are from first half of 2018. ‘Other production’ includes e.g. herds with only sows and no weaners or only weaners without sows or finisher production*.

#### The Questionnaire

The questionnaire addressed the frequency of undertaking various *Salmonella* reducing actions, the perceived effectiveness of the actions, and the perceived costs of the actions. The farmers were presented with 12 possible actions related to management procedures, hygiene and feed changes (corresponding to the actions listed in Table 3). Questions related to the 12 possible actions are shown below (questions 1–4). The questionnaire also included questions about feeding and flooring systems in the herds as these were identified as risk factors (questions 5 and 6). The precise wording for the used questions is shown in Supplementary Material.

*Question 1: You will be introduced to a number of actions that might reduce Salmonella prevalence. For each of the mentioned actions, we ask you to state whether you think that the action has an effect on Salmonella prevalence, the prevalence of other diseases, productivity, or has no effect. You can tick off multiple effects*.

- Response categories: It reduces *Salmonella* prevalence, it reduces prevalence of other diseases, it increases productivity, it has no effect, don't know.

*Question 2: For each of the mentioned actions, we ask you to state whether you have previously or presently implemented that action with the purpose of keeping a low prevalence of Salmonella*.

- Response categories: I am or have previously implemented this action, I have not tried to implement this action.

*Question 3: For each of the mentioned actions, we ask you to state whether you think that action has reduced Salmonella prevalence*.

- Response categories: I think it has an effect (it was not mandatory to answer).

*Question 4: For each action, please state which types of costs you experience or think that you would experience if you implemented the action. You can tick off multiple types of costs for each action*.

- The listed types of costs included: Time costs, lower productivity, running expenses, capital investments, costs of changing habits/cumbersome, requires new knowledge, no particular costs, don't know.

Question 5: Which feeding system is your main system to your finishers?

- Response categories: Home-mixed wet feed restricted quantity, wet feed based on purchased ready-mix restricted quantity, home-mixed dry feed with *ad libitum* quantity, dry feed based on purchased ready-mix with *ad libitum* quantity, other/multiple feeding systems.

Question 6: Which flooring system do you have for your finishers?

- Response categories: Solid floor in more than half of the area (and slatted in the remaining area), solid floor in less than half of the area, combination of drained and slatted floor, other/multiple flooring systems.

In order to keep the questionnaire short and manageable, the 12 actions that might reduce *Salmonella* prevalence were assessed individually thereby implicitly assuming that they are independent (both in terms of being implemented independently and that their effects are independent). This is not in line with practice. As a mitigating circumstance, the aim of the study was to investigate the perceived effects of the actions and not scientifically documented effects. We have assumed that even if effects and costs of actions are not independent, the farmers will be able to form an opinion of the effects and costs of each individual action.

We have not distinguished between actions according to whether they were implemented to prevent a low *Salmonella* prevalence from rising or to reduce a high level of *Salmonella* prevalence. In the first case, *Salmonella* prevalence would be a function of actions taken whereas in the latter case, the actions would be a function of observed *Salmonella* prevalence. Such a distinction can be difficult make in practice and we pondered that distinguishing between actions on this ground would prolong the questionnaire unnecessarily. Consequently, the farmers' responses might reflect a mix of both situations. Hence, a simultaneity issue could be present in the statistical analysis.

#### Methods

Research question RQ2 was investigated using descriptive statistics of Question 1 to Question 4 to document how widespread various beliefs are among pig farmers.

Research question RQ3 was addressed by estimating the relationship between farmers' likelihood of undertaking a given action on the one hand and their perceptions of types of costs, the perceived effect of the action and central herd characteristics on the other hand. The statistical model was based on an empirical operationalization of Equation (4). To carry out the estimations, we assumed that decisions to implement *Salmonella* control actions were based on observing a given *Salmonella* prevalence in the herd. For each *Salmonella* control action, we used a logistic regression approach, where the likelihood (expressed as log-odds ratio) of the action being used (now or in the past) constituted the dependent variable. The following model was estimated for each action:

(5) $$\begin{array}{l} \text{log}\frac{P\left(x=1\right)}{1-P\left(x=1\right)}=\beta_{0}+\beta_{1}\;Think\;effect+\beta_{2}\;Niche\\ \;+\beta_{3}\;S.level2+\beta_{4}\;S.level3+\beta_{5}\;Cost+\beta_{6}\;Effect\\ \;+\beta_{7}\;Feed+\beta_{8}\;Floor+\varepsilon \end{array}$$

The variable *x* is a dichotomous variable assuming the value 1 if the farmer uses or has used the action and 0 otherwise. The parameters β_1_, …β_8_ capture the individual effects on the explanatory variables on the likelihood of implementing the action. The variable *Think effect* assumes the value 1 if the farmer associates a positive effect with the action toward *Salmonella* and 0 otherwise. The variable *Niche* assumes the value 1 if the herd is a breeding herd or has another high quality niche production and 0 otherwise. The variable *S. level 2* assumes the value 1 if the herd has been in *Salmonella* level two within the last 5 years, and *S. level 3* assumes the value 1 if the herd has been in *Salmonella* level three within the last 5 years–and zero otherwise. The variable *Cost* captures eight different types of perceived costs: time costs, reduced productivity, running expenses, investments, costs of changing habits, requires new knowledge, no particular costs, undecided. For each type of cost, the variable assumes the value 1 if the farmer associates that type of cost with the action and 0 otherwise. The variable *Effect* captures three types of perceived effects of the *Salmonella* control: reducing *Salmonella* prevalence, reducing prevalence of other diseases, or increasing productivity. For each type of effect, the variable assumes the value 1 if the farmer associates that effect with the action and 0 otherwise. The variable *Feed* captures the feeding system used in the herd from a list of five possible systems, taking the value 1 if a given feeding system is used and 0 otherwise. The variable *Floor* represents the flooring system used in the herd from a list of four possible types of floor taking the value 1 if a give flooring system is used and zero otherwise. Type of floor and elements of the feeding strategy are considered fixed in the short run but can be included as decision variables in a long run analysis.

The farmers' perceptions of productivity effects are represented in two different ways in the logistic regression. Firstly, one of the dummy variables in *Cost* is “reduced productivity.” Secondly, one of the dummy variables in *Effect* is “it increases productivity.”

The logistic regression analyses were only carried out for eight models of *Salmonella* controls. For four of the actions, there was too little variation. The actions not included are: the use of fermented feed (dry or wet); having high hygiene for workers, visitors, dogs, cats, tools; and rodent control. The logistic regressions were conducted using the software package R.

## Results and Discussion

### Results and Discussions Related to RQ1

The costs related to actions that can be implemented in existing facilities on all farms are presented in Table 5. The actions are presented in ascending cost order per finisher pig. The penalties per pig are also included in Table 5 to ease comparison between costs and benefits of *Salmonella* control actions. For a farmer with costs equal to the industry average costs, several actions could be implemented cheaper than the costs of paying the penalty for being in *Salmonella* level two. The five cheapest actions have aggregated costs lower than the penalty of 2%. Another two actions can be implemented at a lower cost than the 4% penalty. Finally, the last of the actions included in the cost estimation can be implemented at a lower aggregated cost than the 6% penalty. Hence, based on average industry costs, Table 5 indicates that farmers could initiate several actions with lower costs than paying the penalty of 2 or 4%.

**Table 5:** Industry cost estimates for applying on farm actions to reduce *Salmonella* prevalence and penalties for *Salmonella* prevalence (per finisher pig).

**Action**	**Slaughter pigs [ϵ-cent/pig]**	**Aggregated costs [ϵ-cent/pig]**
Have extra rodent control	13.4	
Maintain high hygiene standards	28.2	41.6
Have extra good hygiene before new batches are introduced	33.6	75.2
Use fermented wet feed	40.3	115.5
Use feed with high barley content	94	209.5
*2% penalty*		*258*
Use rough milled feed	120.8	330.3
Use acidified feed	123.5	454
*4% penalty*		*536*
Use acidified drinking water	131.5	585
*6% penalty*		*804*
*8% penalty*		*1,072*

*Detailed information about cost estimates can be found in Table 3*.

Below we illustrate the effects of the penalty scheme at herd level. We provide two examples the size of penalties to be paid at the abattoir for an average farm delivering 10,000 finisher pigs per year (46):

Example 1: Six months in *Salmonella* level two induces a loss in income of 13,000 Euro in penalties.

Example 2: Six months in *Salmonella* level 2 and 6 months in level three induces a loss in income of 40,000 Euro in penalties.

These penalties can be considered as the benefits for a herd of staying in *Salmonella* level one, and thus they constitute the break-even amount to spend on *Salmonella*-reducing actions.

The average profit of a Danish pig farm provides a reference for the significance of the penalties. During the period between 2014 and 2017, a full time farm in Denmark had an average negative profit of 3,100 Euro ranging from minus 52,000 Euro in 2014 to plus 69,000 Euro in 2017. Thereby, the penalty scheme might have a significant effect on the farm economy.

As we do not know the effect of the action, the cost effectiveness is not known.

### Results and Discussions Related to RQ2

With heterogeneity between herds, there might be farmers who do not view the costs and benefits of *Salmonella* control as estimated in Table 5. In order to address the potential variations among farmers, Table 6 presents how widely the various actions are or have been used together with farmers' perceptions of the effectiveness of the actions.

**Table 6:** Share of farmers who use/have used the presented *Salmonella* reducing action and the share of farmers who think it has an effect (in percent of respondents).

	**Being used**	**I think it has an effect**
Buy pigs from herds with lowest *Salmonella* level	55	23
All in-all out	80	28
Have extra good hygiene between batches	91	23
Use acidified feed	74	29
Use fermented dry feed	9	11
Use fermented wet feed	16	16
Use rough milled feed	55	19
Use feed with high content of barley	61	18
Use home mixed feed rather than ready made	62	20
Use acidified drinking water	68	22
Have high hygiene for workers, visitors etc.	84	20
Have extra rodent control	95	21

*One hundred and forty eight of the producers answered both questions and the shares shown in the table are shares out of 148 producers*.

Table 6 shows that most of the presented actions have been used widely in Danish pig farms. The hygiene-related actions are used by the vast majority of the farmers (more than 80% of the respondents) while acidification of feed or drinking water are used by three out of four of the respondents. The actions with the lowest uptake among farmers include fermented dry feed (used by 9% of the respondents) and fermented wet feed (used by 16% of the respondents). We also find that less than one third of the farmers believe that the individual actions are efficacious.

Differences among farmers regarding which control actions they use or have used might be related to the types of costs that farmers associate with the individual actions. Table 7 sheds light on how perceptions of different types of costs associated with the 12 potential *Salmonella* reducing actions are distributed across farmers. Some farmers find it cumbersome to implement new actions due to change of habits (“Habits” in Table 7) and others associate costs with the need to obtain new knowledge to implement actions (“Knowledge” in Table 7). There are also farmers who do not associate particular costs with implementing new actions (“No particular” in Table 7).

**Table 7:** Share of farmers who associate various types of costs with the presented *Salmonella* reducing actions (in percent of respondents).

**Action**	**Time costs**	**Loss of productivity**	**Running expenses**	**Investment**	**Habits**	**Knowledge**	**No particular**	**Don't know**
Buy pigs from herds with low *Salmonella*	2	2	26	8	4	2	39	26
All in-all out system	20	15	13	11	14	2	46	7
Extra good hygiene between batches	55	2	25	5	26	1	32	4
Acidification of feed	4	2	82	7	4	1	5	12
Fermented dry feed	2	1	23	12	1	4	1	64
Fermented wet feed	5	1	22	18	2	4	5	56
Rough milled feed	1	42	35	2	2	1	17	24
Feed with high content of barley	1	30	26	2	1	1	25	26
Home-mixed feed	23	1	15	38	12	5	23	20
Acidification of drinking water	17	3	73	17	12	1	6	15
High hygiene for workers, visitors etc.	30	1	14	6	14	2	54	10
Rodent control	22	1	64	2	11	1	23	6

*One hundred and forty two of the producers answered this question*.

An important observation from Table 7 is that many of the farmers answered “Don't know” to the question of which costs they associated with the individual control actions. This results indicates that many farmers have not really thought about the costs which is potentially surprising given that many of the farmers have used the actions. Also, all actions have in common that only a small minority of the farmers perceive them as costly in terms of knowledge acquisition.

We observed differences across actions as well. For some of the actions, the associated costs are consistent with the expert-estimated cost described in Tables 3, 5. The use of rough milled feed and higher barley content in the feed are associated with lower productivity by the farmers as well as by the experts. Also, the associations of running costs with acidified feed and drinking water as well as with rodent control are in line with expert opinions. Another interesting observation is that for four of the actions, more than 30% of respondents did not associate implementation with extra costs. The four actions were “buy pigs from herds with low *Salmonella* level,” “all in–all out” and the two hygiene-related actions. Around 20% of the respondents found no particular costs associated with the feed-related actions rough milled feed, feed with high content of barley and using home-mixed feed. As other farmers associated these actions with extra costs, our results indicate that actions are perceived to have very different costs across herds.

Differences in farmers' perceptions of the effects of various control actions and their costs might also be related to differences in disease pressure where management and hygiene actions might affect other diseases. Farmers' perceptions of whether the individual actions have an effect on *Salmonella* level, other diseases, productivity, or no effect is shown in Table 8.

**Table 8:** Farmers' perception (in percent of respondents) of whether the individual actions have an effect on *Salmonella* level, other diseases, productivity, or no effect.

**Action**	**Reduces *Salmonella* prevalence**	**Reduces other diseases**	**Increases productivity**	**No perceived effects**	**Don't know**
Buy pigs from herds with low *Salmonella*	72	15	21	9	13
All in-all out/systematic shifting of batches	72	43	48	5	7
Extra good hygiene when new batches are introduced	79	48	47	3	3
Acidification of drinking water	84	17	21	4	15
Acidification of feed	15	2	6	8	71
Fermented dry feed	28	7	10	6	63
Fermented wet feed	60	22	4	8	20
Rough feed	48	20	7	9	34
Feed with high content of barley	53	11	11	5	35
Home-mixed feed instead of ready made feed	83	13	10	3	13
High hygiene for workers, visitors etc.	64	46	26	5	11
Rodent control	82	40	21	3	9

*A total of 149 farmers answered this question*.

For all actions, we note that only a small part of the farmers (between 3 and 9%) believes the action has no positive effect on either reducing the prevalence of *Salmonella* or of other diseases or on increasing productivity. All actions are associated with all types of effect to various degrees. All actions score high on their effect on *Salmonella* reduction. The only exception is fermented feed where less than one third of the sampled pig farmers believe it reduces *Salmonella* risk. Comparing the results presented in Tables 6, 8 reveals that significantly more farmers state the 12 listed actions to have a reducing effect on *Salmonella* prevalence (Table 8) than farmers stating that they have tried to implement the actions and that they believe that the actions have an effect (Table 6). Unfortunately, it is not possible to dig deeper into these differences where possible explanations could be the different wordings of the two questions and the order of the questions. Unexpectedly many “don't know” answers were found for fermentation-related actions (two out of three responses). This could indicate a high level of confusion about the question formulation or the action itself–or both.

Addressing RQ2, the most significant result is that less than one third of the farmers believe that the actions they have implemented to reduce *Salmonella* prevalence have had an effect. Hence, the perceived benefits of the *Salmonella* control actions are rather weak. A potential explanation, inspired by a respondent's comment to an open question in the questionnaire, was that as they often initiate multiple actions at the same time, they do not know which of the actions are effective. Secondly, an important result regarding perceived costs of *Salmonella* control actions is that running expenses are widely associated with all 12 listed actions. Thirdly, the feed-related actions regarding high content of barley or roughly milled feed are in particular associated with loss of productivity. Fourthly, many farmers in the sample link management-related actions and hygiene-related actions with not only reducing *Salmonella* prevalence but also with reducing the prevalence of other diseases as well as with increasing productivity. As a contrast, feed-related actions are mainly associated with reducing *Salmonella* prevalence.

### Results and Discussions Related to RQ3

Potential explanatory factors for farmers' choice of using the listed *Salmonella* control actions are shown in Table 9. At the risk of information overload, we have included significant as well as in-significant variables from the regression analysis in Table 9. An advantage of keeping all explanatory variables in the model include that it eases comparison between actions and that the sign of an insignificant factor also provides information about weak effects that with a larger data set might turn out significant.

**Table 9:** Logistic regression model explaining the choice of *Salmonella* control with variables related to perceptions of costs and effects, *Salmonella* level in the herds as well as herd characteristics.

	**Buy pigs**	**All in-all out**	**Hygiene new batch**	**Acid in feed**	**Rough feed**	**Barley feed**	**Home mix feed**	**Acid in water**
Constant	1.72 (1.95)	0.46 (1.81)	8.40* (4.00)	−20.3 (2,855)	−2.29 (1.27)	−2.85 (1.52)	−1.62 (1.41)	−4.07 (2.33)
**Parameter estimates related to perceived types of costs (standard errors)**								
Time costs	21 (1,471)	1.13 (1.19)	−1.41 (1.92)	−3.53* (1.69)	18.25 (6,523)	13.69 (3,378)	0.57 (0.93)	3.72** (1.34)
Productivity costs	1.66 (2.18)	−1.43 (1.21)	−42.38 (24,244)	−0.84 (3.29)	−0.31 (0.8)	0.57 (1.11)	0.88 (2.24)	−1.93 (1.76)
Running expenses	−2.07 (1.75)	−0.71 (1.3)	−1.35 (1.98)	17.93 (2,855)	0.29 (0.74)	1.38 (1.05)	0.53 (1.15)	−2.65 (1.81)
Investments	−2.89. (1.72)	−2.08 (1.29)	21.29 (5,115)	17.5 (1,931)	−16.32 (3,425)	16.09 (3,010)	−1.18 (1.19)	−2.29* (0.94)
Habits/ cumbersome	−19.04 (1,471)	0.79 (1.48)	−2.53 (1.78)	−2.44 (1.68)	34.9 (4,844)	17.34 (3,378)	1.06 (1.09)	−0.77 (1.42)
Knowledge	1.91 (2.4)	17.37 (1,947)	18.89 (10,220)	2.33 (4,272)	−23.34 (9,840)	15.13 (3,378)	−0.35 (1.72)	−1.46 (14.53)
No particular costs	−0.73 (1.58)	0.78 (1.28)	−2.18 (2.03)	18.41 (2,855)	−0.09 (1.06)	1.16 (1.28)	0.3 (1.38)	15.81 (1,620)
Don't know about costs	−3.74* (1.7)	−0.81 (1.5)	−4.39 (2.92)	16.1 (2,855)	−2.40* (1.03)	−1.12 (1.28)	−1.29 (1.25)	−4.25* (1.9)
**Parameter estimates related to perceived effects (standard errors)**								
Effect on *Salmonella*	1.62** (0.61)	2.10* (0.93)	3.37 (2.04)	2.7* (1.07)	1.82** (0.6)	2.22*** (0.59)	1.73* (0.68)	3.16** (1.05)
Effect on other diseases	0.45 (0.83)	−0.26 (0.95)	−2.16 (1.91)	0.2 (1.1)	0.22 (0.71)	0.35 (0.7)	1.13 (1.19)	5.43* (2.19)
Increases productivity	1.19 (0.82)	3.34** (1.29)	5.56* (2.3)	2.84* (1.33)	19.67 (2,267)	3.07* (1.33)	1.26 (1.22)	−0.80 (1.31)
I think it has an effect	−2.56*** (0.77)	−4.09*** (1.09)	−4.08* (1.93)	−1.82* (0.76)	−2.46** (0.78)	−2.89*** (0.84)	−1.04 (0.82)	−0.89 (0.97)
**Parameter estimates related to** ***Salmonella*** **level and production system (standard errors)**								
Level 2, past 5 yrs.	0.54 (0.71)	−0.33 (0.89)	−3.83 (2.48)	2.27** (0.78)	0.83 (0.7)	0.14 (0.74)	2.96** (1.03)	3.27** (1.06)
Level 3, past 5 yrs.	1.16 (0.87)	−0.12 (1.07)	−3.84 (2.71)	2.57** (0.89)	1.64* (0.81)	0.18 (0.79)	2.26* (0.94)	3.78** (1.19)
Breeding herd	−1.36 (1.07)	1.60 (1.40)	18.12 (3,712)	−0.57 (1.02)	4.56** (1.57)	2.29 (1.17)	0.10 (1.12)	1.61 (1.37)
Home mixed dry feed	−1.56* (0.76)	−2.31 (1.2)	−1.68 (2.16)	−0.50 (1.02)	−0.25 (0.67)	1.13 (0.72)	1.62 (1.24)	0.51 (0.87)
Other/multiple feeding systems	0.44 (1.23)	1.85 (1.93)	−4.40 (3.46)	2.06 (1.53)	0.37 (1.25)	−1.40 (1.09)	−1.27 (1.33)	−0.34 (1.33)
Wet feed, purchased	2.14 (1.34)	−1.94 (1.58)	−3.82 (2.97)	0.70 (1.23)	0.29 (1.22)	1.49 (1.37)	−1.19 (1.17)	4.02* (1.74)
Dry feed, purchased	0.67 (0.71)	−1.01 (1.08)	−3.67 (2.23)	−1.18 (0.96)	1.66* (0.70)	0.09 (0.7)	−3.27*** (0.9)	2.61* (1.04)
Special production	−0.78 (0.62)	2.84* (1.16)	0.32 (1.50)	−0.12 (0.82)	0.37 (0.62)	0.75 (0.71)	0.79 (0.87)	0.37 (0.84)
Max 50% solid floor	−1.36 (0.79)	0.53 (0.97)	0.93 (1.67)	0.63 (0.92)	0.54 (0.76)	2.27* (0.88)	1.32 (0.9)	0.53 (0.96)
Combi–floor drained/slatted	−0.47 (0.72)	1.65 (0.9)	2.00 (1.65)	0.98 (0.85)	0.5 (0.69)	1.31 (0.73)	0.94 (0.87)	2.07* (0.92)
Other/multiple flooring	0.51 (1.06)	−1.78 (1.4)	18.64 (3,445)	0.01 (1.37)	1.06 (0.93)	2.16 (1.16)	0.44 (1.46)	2.83 (1.59)

The dependent variables are the “Being used (now or in the past).” Significance levels: <0.05 *; <0.01**; <0.001***.

A noteworthy result is that none of the variables related to perceived types of costs had a significant effect on whether an action was used. The only exception being that time costs had a negative effect on the use of acidified feed (as could be expected) but a positive effect on the use of acidified water (not as could be expected).

Having been in *Salmonella* level two or three had a significant and positive effect on the likelihood of using feed-related actions and of using acidified water. Believing that an action increased productivity also had a positive effect on all actions and significant for several. Only exception was the use of acid in water where a belief in increased productivity had a negative but in-significant effect.

As expected, across actions, a belief that an action had a *Salmonella* reducing effect had a significant and positive effect on the probability of using it. The only exception was increased hygiene between batches, where its use was not affected by whether the farmer perceived it to have a *Salmonella* reducing effect. We suggest that this result indicates that the farmers have other reasons than *Salmonella* concerns to increase hygiene between batches.

Farmers with breeding herds are more prone than other farmers to use all means to reduce *Salmonella* including actions estimated to be more expensive such as rough feed (see Table 3). This result is as expected as the costs to the breeding herds of not being able to sell their breeding animals are high. Thereby, breeding farmers have stronger incentives than other farmers to reduce *Salmonella* in their herds. Another statistically significant result is that farmers with special production are more prone to use the action “all in/all out,” which is most likely due to a correlation between farmers who have special production also have barns that makes batch production relevant.

Less expected, we found that for most actions, the variable “I think it has an effect” has a negative and significant association with the decision to implement the action. We stress that we cannot draw conclusions on causality, but we would have expected the opposite, namely a positive correlation between a belief in that it has an effect and choosing an action. We have two possible explanations for the negative correlation. One reason for the negative correlation is that there is a great deal of confusion about the effects of the individual actions because multiple actions are often implemented simultaneously. Another possible reason is linked to the lack of time line in the data. Rather than looking at the result as farmers implementing a *Salmonella* control action even though they believe it does not have an effect, we could interpret the result as showing that farmers who are or have been implementing a given action have experienced that it does not reduce *Salmonella* prevalence. As the data do not allow us to distinguish between farmers who are implementing a given action and farmers who have done so in the past and thereby have an experience regarding its effectiveness, we cannot dig more into that possible reason (we thank an anonymous referee for suggesting this association). Further studies, possibly involving detailed interviews are needed to dig deeper into this apparent paradox.

Addressing RQ3, the overall picture was that perceptions of costs and effects and herd characteristics had a non-systematic effect on the implementation of 12 listed *Salmonella* reducing actions. A few central effects are nevertheless worth highlighting. First, farmers who believe that an action has a *Salmonella-*reducing effect or that it increases productivity are more likely than other farmers to choose that particular action. This suggests that the expected effectiveness of the action either on *Salmonella* prevalence or productivity is an important determinant for the choice of action. Also, we found that farmers who have been in *Salmonella* level two or three within the last 5 years were more prone to use acidified feed, acidified drinking water, and home mixed feed.

## General Discussion

The aim of the study was to investigate whether the Danish *Salmonella* action plan — and in particular the penalty scheme — had an effect on pig farmers' efforts to control *Salmonella* in their herds. The estimated average costs and estimated benefits of *Salmonella* control together with the observation that the period in which the farmers stay in level two or three is rather short (2–3 months), leads us to conclude that the pig farmers are provided with economic incentives to implement actions aiming to reduce *Salmonella* prevalence. Further, when the farmers were asked, they expected an increase in the general prevalence of *Salmonella* if the penalty scheme were terminated (47).

**Figure 1:**
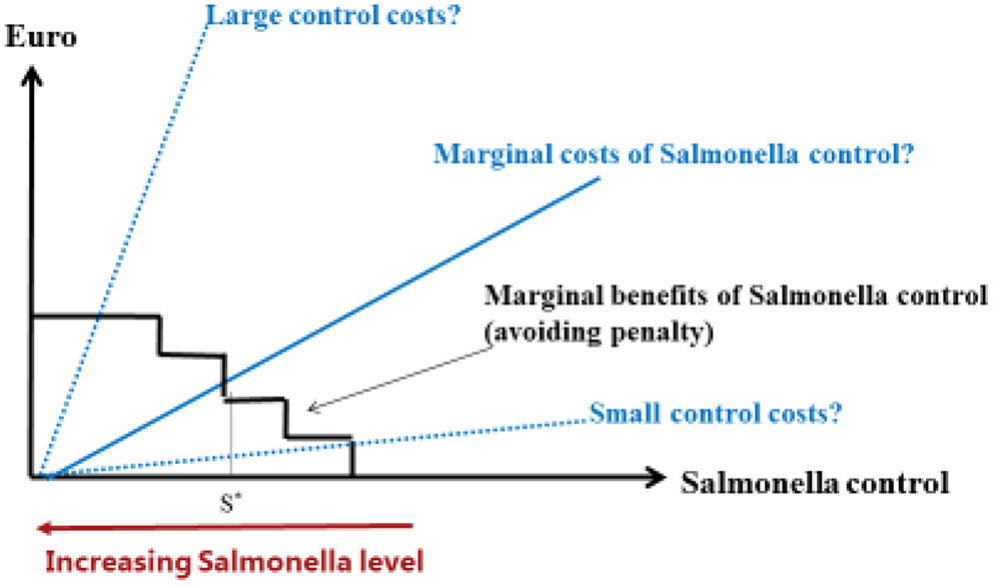
Illustration of optimal *Salmonella* control problem with uncertainty about costs of *Salmonella* control.

This study revealed large variations between farmers regarding perceived effects and perceived costs of *Salmonella*-reducing actions. Therefore, from a policy design perspective, the approach with freedom to choose which action to implement is preferred over mandatory *Salmonella* control actions. On the other hand, the uncertainty surrounding the perceived costs and effectiveness of the individual actions places a great deal of risk on the farmers' shoulders. Figure 1 illustrates a theoretical case with increasing marginal costs and decreasing marginal benefits of *Salmonella* control. It highlights the difficulty for a farmer to optimize *Salmonella* control when effects of actions and thereby costs of the actions are uncertain. The optimal level of *Salmonella* control is given by the intersection between the marginal benefit curve (represented by the downward sloping laddered curve) and the upward sloping marginal cost curve, which is positioned somewhere between the “small control costs” and “large control costs” curves. When the position of the marginal cost curve is uncertain, it becomes difficult to determine the optimal level of control.

Based on the heterogeneity in farmers' perceptions of costs and effects and the fact that farm characteristics vary with respect to e.g., feeding system and eligibility of section-wise production, we claim that individual herd effects are important to acknowledge when regulating a zoonosis like *Salmonella*. One of our puzzling results is that it is more likely for farmers who have not implemented a given *Salmonella* control action to believe that the action has an effect on *Salmonella*. This could indicate that farmers who have used the actions are less convinced of the effectiveness, and/or that implementation of the actions may have been driven by other motives than *Salmonella* control.

The logistic regression analysis indicated that farmers who have used a specific action were more likely to believe that this action increases productivity. This result reflects that the perceived net costs of implementing an action are lower than the running expenses (as they are adjusted for productivity gains), and hence that effect b from the theoretical model tends to be important. Direct and indirect costs of the actions (effect c from the theoretical model) tends to have a negative – but statistically insignificant – effect on farmers' propensity to implement the actions.

Due to the high uncertainty about costs and effects of the individual actions, farmers might find it optimal to use only low net cost actions and then hope this is effective enough to stay out of *Salmonella* level two or three, or even being willing to accept to have infrequent and short time periods with high prevalence. We have not addressed the important time dimension in the farmer's decision problem. However, it is likely that actions in most cases are only necessary to implement for a shorter period for the *Salmonella* level to decrease. Therefore, it is unlikely that farmers in the longer run are better off with permanent penalties compared to using reducing actions for a shorter time period. A limiting aspect of the analysis is that we have mainly included control actions that can be applied to temporary interventions to reduce *Salmonella* prevalence. A different set of questions that could address the strategic considerations of disease management in herd management will be valuable to include in a future study. The importance of veterinarians is emphasized in a report by Olsen and Christensen (47), where eight farmers out of 10 state that they base their choice of *Salmonella* control on advice from their veterinarian.

We suggest that an important task for future research is to combine studies aiming at investigating which action is most effective to lower the *Salmonella* infection level when pigs enter the abattoir with social science studies as ours investigating to what extent pig farmers have sufficient economic incentives and other motivations to control in-herd *Salmonella* prevalence. Also, valuable information could be obtained in future studies where more details about potential differences in perceptions and in *Salmonella* control practices across different types of production.

## Conclusion

We found that Danish farmers are provided with economic incentives to reduce *Salmonella* prevalence at the herd level – as a consequence of the relation between the estimated costs (industry averages) and the estimated benefits of *Salmonella* control in terms of avoided penalties. The farmers' link a variety of costs with *Salmonella* control but it was noteworthy that variations in perceived costs could not explain the farmers' choice of *Salmonella* reducing actions. The hygiene and management related actions are not only implemented to reduce *Salmonella* prevalence in pig herds and are likely to be maintained even with a removal of the penalty scheme. On the other hand, feed and water related actions are mainly motivated by *Salmonella* reductions and are more likely to be discarded without a penalty scheme. While the incentives provided by the present action plan and in particular the penalty scheme are sound, the uncertainty about costs and effects of *Salmonella* control actions hamper the effectiveness of the penalty scheme as a regulatory tool.

## Data Availability Statement

The raw data supporting the conclusions of this article will be made available by the authors, without undue reservation.

## Author's Note

The article is based on research carried out in 2018 and 2019 by two of the authors for the Danish Ministry of Environment and Food, with the aim to evaluate the Salmonella reducing effects of an existing penalty scheme for Danish finisher pigs. The work was followed by a reference group with representatives from the Danish pig industry, The Danish Veterinary and Food Administration, Technical University of Denmark (National Veterinary Institute and National Food Institute).

## Author Contributions

JO and TC has contributed to conceptualization, data curation, formal analysis, validation, writing of the original manuscript, editing, and writing of the final manuscript. JD has contributed to conceptualization, formal analysis, validation, writing of the original manuscript, editing, and writing of the final manuscript. All authors contributed to the article and approved the submitted version.

## Conflict of Interest

JO and TC collaborate with employees from the Danish pig industry in a separate project with no links to the project here reported. The remaining author declares that the research was conducted in the absence of any commercial or financial relationships that could be construed as a potential conflict of interest.

## References

[1] EFSA and ECDC. (European Food Safety Authority, & European Centre for Disease Prevention and Control) The European Union one health 2018 zoonoses report. EFSA J. (2019) 17:e05926. 10.2903/j.efsa.2019.5926 32626211 PMC7055727

[2] Bonardi S. *Salmonella* in the pork production chain and its impact on human health in the European Union. Epidemiol Infection. (2017) 145:1513–26. 10.1017/S095026881700036X 28241896 PMC9203350

[3] Alban L Stärk KDC. Where should the effort be put to reduce the *Salmonella* prevalence in the slaughtered swine carcass effectively? Prevent Vet Med. (2005) 68:63–79. 10.1016/j.prevetmed.2005.01.001 15795016

[4] Jensen JD Belay DG. Produktions- og Sektorøkonomiske Konsekvenser af at Reducere Antibiotikaforbruget i Dansk Svineproduktion, IFRO Commissioned work (2019).

[5] Pigou AC. The Economics of Welfare. 4th edition. London: Macmillan (1932).

[6] EU. Regulation no 2160/2003 of the European Parliament and of the Council of 17 November 2003 on the Control of Salmonella and Other Specified Food-Borne Zoonotic Agents. (2003). Available online at: https://eur-lex.europa.eu/legal-content/EN/TXT/PDF/?uri=CELEX:32003R2160&from=EN (accessed April 4, 2021).

[7] Creus E. Swine Salmonellosis Surveillance and Control Programmes in the EU: General Basis. (2014). Available online at: https://www.pig333.com/articles/swine-salmonellosis-surveillance-and-control-programmes-in-the-eu_8709/ (accessed April 4, 2021).

[8] Danish Veterinary and Food Administration. Salmonella og svin. (2020). Available online at: https://www.foedevarestyrelsen.dk/Leksikon/Sider/Svinog-Salmonella.aspx (accessed April 4, 2021).

[9] Baptista FM Halasa T Alban L Nielsen LR. Modelling food safety and economic consequences of surveillance and control strategies for *Salmonella* in pigs and pork. Epidemiol Infect. (2011) 139:754–64. 10.1017/S0950268810001767 20653990

[10] Alban L Baptista FM Møgelmose V Sørensen LL Christensen H Aabo S. *Salmonella* surveillance and control for finisher pigs and pork in Denmark — a case study. Food Res Int. (2012) 45:656–65. 10.1016/j.foodres.2011.02.050

[11] Goldbach SG Alban L. A cost–benefit analysis of Salmonella-control strategies in Danish pork production. Preventive Veterinary Medicine. (2006) 77:1–14. 10.1016/j.prevetmed.2005.10.008 16879887

[12] Lawson LG Jensen JD Christiansen P Lund M. Cost-effectiveness of *Salmonella* reduction in Danish abattoirs. Int J Food Microbiol. (2009) 134:126–32. 10.1016/j.ijfoodmicro.2009.03.024 19427047

[13] Botteldoorn N Heyndrickx M Rijpens N Grijspeerdt K Herman L. *Salmonella* on pig carcasses: positive pigs and cross contamination in the slaughterhouse. J Appl Microbiol. (2003) 95:891–903. 10.1046/j.1365-2672.2003.02042.x 14633017

[14] Baptista FM Dahl J Nielsen LR. Factors influencing *Salmonella* carcass prevalence in Danish pig abattoirs. Prevent Vet Med. (2010) 95:231–8. 10.1016/j.prevetmed.2010.04.007 20537741

[15] Rostagno MH Callaway TR. Pre-harvest risk factors for *Salmonella* enterica in pork production. Food Res Int. (2012) 45:634–40. 10.1016/j.foodres.2011.04.041

[16] Danish Veterinary and Food Administration. Salmonella Handlingsplan for Svin SH5. (2013). Available online at: https://www.foedevarestyrelsen.dk/SiteCollectionDocuments/25_PDF_word_filer%20til%20download/04kontor/Mikro%20zoonose/Salmonellahandlingsplan%20svin%20SH5.pdf (accessed April 4, 2021).

[17] Ministry of Environment and Food of Denmark. Executive Order 1792 from December 2, 2020. Bekendtgørelse om salmonella hos svin (2020).

[18] SEGES. Vejledning til Svineproducenter om Salmonella. Pig Research Centre, Danish Agriculture and Food Council. (2021). Available online at: https://svineproduktion.dk/-/media/PDF/Pjecer/Vejledning-til-svineproducenter-om-Salmonella.ashx (accessed April 4, 2021).

[19] Ministry of Environment and Food of Denmark. Guide 9697 from August 7, 2017. Vejledning til bekendtgørelse om salmonella hos svin (2017).

[20] Simon-Grife M Martín-Valls GE Vilar MJ García-Bocanegra I Martín M Mateu E. Biosecurity practices in Spanish pig herds: perceptions of farmers and veterinarians of the most important biosecurity measures. Prevent Vet Med. (2013) 110:223–31. 10.1016/j.prevetmed.2012.11.028 23273732

[21] Gotter V Klein G Koesters S Kreienbrock L Blaha T Campe A. Main risk factors for *Salmonella*-infections in pigs in north-western Germany. Prevent Vet Med. (2012) 106:301–7. 10.1016/j.prevetmed.2012.03.016 22534071

[22] Wilhelm B Rajić A. Parker S Waddell L Sanchez J Fazil A. Assessment of the efficacy and quality of evidence for five on-farm interventions for Salmonella reduction in grow-finish swine: a systematic review and meta-analysis. Prevent Vet Med. (2012) 107:1–20. 10.1016/j.prevetmed.2012.07.011 22921852

[23] Bjork KE Fields V Garber LP Kopral CA. Factors associated with *Salmonella* prevalence in U.S. Swine grower–finisher operations. Foodborne Pathog Dis. (2018) 15:489–97. 10.1089/fpd.2017.2364 29762053

[24] Jørgensen L Dahl J Wingstrand A. The effect of feeding pellets, meal and heat treatment on the *Salmonella* prevalence in finishing pigs. In: Proceedings of the 2nd International Symposium on the Epidemiology and Control of Salmonella. Pork, WA (1999) 10.31274/safepork-180809-1034

[25] Kjeldsen N Dahl J. The effect of feeding non-heat treated, non-pelleted feed compared to feeding pelleted, heat-treated feed on the *Salmonella*-prevalence of finishing pigs. In: Proceedings of the 2nd International Symposium on the Epidemiology and Control of Salmonella. Pork, WA (1999). 10.31274/safepork-180809-1035

[26] Jørgensen L Kjaersgaard H Wachmann H Jensen B Knudsen K. Effect of wheat bran and wheat:barley ratio in pelleted feed on *Salmonella* prevalence and productivity of finishers. In: Proceedings of the 4th International Symposium on the Epidemiology and Control of Salmonella and Other Foodborne Pathogens. Pork (2001). 10.31274/safepork-180809-209

[27] Jørgensen L Boes J Kranker S Kjaersgaard H Wachmann H. Effect of an optimised pelleted diet on Salmonella prevalence and pig productivity. In: Fifth International Symposium on the Epidemiology and Control of Foodborne Pathogens in Pork. Crete (2003). 10.31274/safepork-180809-480

[28] Lucia ADD Ostanello F. On-farm risk factors associated with *Salmonella* in pig herds. Large Animal Rev. (2020) 26:133–40. Available online at: https://www.largeanimalreview.com/index.php/lar/article/view/124/79

[29] Creus E Pérez JF Peralta B Baucells F Mateu E. Effect of acidified feed on the prevalence of *Salmonella* in market-age pigs. Zoonoses Pub Health. (2007) 54:314–9. 10.1111/j.1863-2378.2007.01069.x 17894642

[30] Andres VM Davies RH. Biosecurity measures to control *Salmonella* and other infectious agents in pig farms: a review. Comprehen Rev Food Sci Food Safety. (2015) 14:317–35. 10.1111/1541-4337.12137

[31] Farzan A Friendship RM Dewey CE Warriner K Poppe C Klotins K. Prevalence of *Salmonella* spp. on Canadian pig farms using liquid or dry-feeding. Prevent Vet Med. (2006) 73:241–54. 10.1016/j.prevetmed.2005.09.003 16202460

[32] van der Heijden M Dam Niewerth HVD Frankena K. Effectiveness of *Salmonella* control strategies in fattening pigs. In: Sixth International Symposium on the Epidemiology and Control of Foodborne Pathogens in Pork. Rohnert Park, CA (2005). 10.31274/safepork-180809-736

[33] Schmidt PL O'Connor AM McKean JD Hurd HS. The association between cleaning and disinfection of lairage pens and the prevalence of *Salmonella* enterica in swine at harvest. J Food Protect. (2004) 67:1384–8. 10.4315/0362-028X-67.7.1384 15270490

[34] Leirs H Lodal J Knorr M. Factors correlated with the presence of rodents on outdoor pig farms in Denmark and suggestions for management strategies. NJAS Wageningen J Life Sci. (2004) 52:145–61. 10.1016/S1573-5214(04)80010-1

[35] Alarcon P Wieland B Mateus A Dewberry C. Pig farmers' perceptions, attitudes, influences and management of information in the decision-making process for disease control. Prevent Vet Med. (2014) 116:223–42. 10.1016/j.prevetmed.2013.08.004 24016600

[36] Marier E Piers Smith R Ellis-Iversen J Watson E Armstrong D Hogeveen H. Changes in perceptions and motivators that influence the implementation of on-farm *Salmonella* control measures by pig farmers in England. Prevent Vet Med. (2016) 133:22–30. 10.1016/j.prevetmed.2016.09.009 27720024

[37] Lund M Gülec Ö. Kvæg- og Svineproducenters Holdninger til Smittebeskyttelse og Smittebeskyttelsesplaner. IFRO Commission Work 2013/16. Department of Food and Resource Economics, University of Copenhagen. (2013). Available online at: https://static-curis.ku.dk/portal/files/95167529/IFRO_Udredning_2013_16.pdf (accessed April 4, 2021). [In Danish].

[38] Danish Veterinary and Food Administration. The Central Husbandry Register (CHR). Ministry of Environment and Food, Danish Veterinary and Food Administration. (2019). Available online at: https://www.foedevarestyrelsen.dk/english/Animal/AnimalHealth/Central_Husbandry_Register/Pages/default.aspx [In Danish].

[39] Danish Veterinary and Food Administration. The Zoonosis Register. Ministry of Environment and Food, Danish Veterinary and Food Administration (2019). Available online at: https://zoor.fvst.dk/zoor/faces/home?_adf.ctrl-state=41xjcq3vy_3

[40] Sloth NM Tybirk P Dahl J Christensen G. Effekt af Formalingsgrad og Varmebehandling/Pelletering på Mavesundhed, Salmonella-Forebyggelse og Produktionsresultater hos Slagtesvin. Danish Pig Research Centre (1998).

[41] Hansen CF Dahl J Jørgensen L. Effekt af Myresyre i Drikkevand på Forekomst af Salmonella hos Slagtesvin. Danish Pig Research Centre (1999).

[42] Busch ME Jensen T. Smitteafbrydelse og produktivitet i slagtesvinehold i multisite-systemer. Meddelelse Nr. 708: Landsudvalget for Svin, Videncenter for Svineproduktion, Den Rullende Afprøvning. (2005). Available online at: https://svineproduktion.dk/publikationer/kilder/lu_medd/2005/708 (accessed April 4, 2021).

[43] Pedersen AØ Canibe N. Fermentering af Korn Giver en Lille Stigning i Energiværdien. Danish Pig Research Centre (2011).

[44] Jørgensen T. Personal communication, Copenhagen, (2019).

[45] Statistics Denmark. Priser for Jordbrugets Produktionsfaktorer Efter Produkt og Enhed. (2019). Available online at: https://www.statistikbanken.dk/LPRIS35.

[46] SEGES. Få et Overblik Over Landbrugets Indtjening. Landbrugsinfo, Økonomi og ledelse. (2018). Available online at: https://www.landbrugsinfo.dk/oekonomi/oekonomiske-analyser/driftsresultater-priser-prognoser/sider/startside.aspx (accessed April 4, 2021).

[47] Olsen JV Christensen T. Analyse af effekten af det økonomiske incitament i bodssystemet i salmonellahandlingsplanen for svin, 81 s. Copenhagen: Department of Food and Resource Economics (IFRO) Commissioned work 2019/13 (2019).

