# Supplementary material for: Pig Farmers' Perceptions of Economic Incentives to Control Salmonella Prevalence at Herd Level
Source: Front Vet Sci. 2021 Apr 16;8:647697. doi: 10.3389/fvets.2021.647697 (PMC8086553; doi:10.3389/fvets.2021.647697)
Supplement: Supplementary file 1 [file Data_Sheet_1.docx]

**Supplementary material**

Exact formulation of the 6 questions used in the analyses (translated from Danish to English)

*Question 1:*

*You will be introduced to a number of actions that might reduce Salmonella prevalence. For each of the mentioned actions, we ask you to state whether you think that the action has an effect on Salmonella prevalence, the prevalence of other diseases, productivity, or has no effect. You can tick off multiple effects.*

|  | Reduces *Salmonella* prevalence | Reduces the prevalence of other diseases | Increases productivity | No effect | Don’t know |
| --- | --- | --- | --- | --- | --- |
| Buy pigs from herds with low *Salmonella* level |  |  |  |  |  |
| All in-all out / systematic shifting of batches |  |  |  |  |  |
| Extra good hygiene when new batches are introduced |  |  |  |  |  |
| Feed with organic acid |  |  |  |  |  |
| Using fermented dry feed |  |  |  |  |  |
| Using fermented wet feed |  |  |  |  |  |
| Rough milled feed |  |  |  |  |  |
| Feed with high barley content |  |  |  |  |  |
| Home mixed feed |  |  |  |  |  |
| Acidified drinking water |  |  |  |  |  |
| High hygiene for workers, visitors, dogs, cats, tools |  |  |  |  |  |
| Rodent control |  |  |  |  |  |

Questions 2 and 3 were presented in the same table in the questionnaire.

*Question 2 and 3:*

*For each of the mentioned actions, we ask you to state whether you have previously or presently implemented that action with the purpose of keeping a low prevalence of Salmonella – and whether you think that the action has an effect.*

(Note: the respondents had to tick off one of the two first categories but the last category was not mandatory to tick off)

|  | I have or have previously implemented this action | I have not tried to implement this action | I think it has an effect |
| --- | --- | --- | --- |
| Buy pigs from herds with low *Salmonella* level |  |  |  |
| All in-all out / systematic shifting of batches |  |  |  |
| Extra good hygiene when new batches are introduced |  |  |  |
| Feed with organic acid |  |  |  |
| Using fermented dry feed |  |  |  |
| Using fermented wet feed |  |  |  |
| Rough milled feed |  |  |  |
| Feed with high barley content |  |  |  |
| Home mixed feed |  |  |  |
| Acidified drinking water |  |  |  |
| High hygiene for workers, visitors, dogs, cats, tools |  |  |  |
| Rodent control |  |  |  |

*Question 4:*

*For each action, please state which types of costs you experience or think that you would experience if you implemented the action. You can tick off multiple types of costs for each action.*

As the response categories do not fit into the table here, we have replaced the categories with numbers. Note however, that in the original questionnaire, the categories were shown directly in the table. The listed types of costs included: 1) Time costs 2) Lower productivity 3) Running expenses 4) Capital investments 5) Cumbersome to implement 6) New knowledge 7) No particular costs 8) Don’t know.

|  | 1 | 2 | 3 | 4 | 5 | 6 | 7 | 8 |
| --- | --- | --- | --- | --- | --- | --- | --- | --- |
| Buy pigs from herds with low *Salmonella* level |  |  |  |  |  |  |  |  |
| All in-all out / systematic shifting of batches |  |  |  |  |  |  |  |  |
| Extra good hygiene when new batches are introduced |  |  |  |  |  |  |  |  |
| Feed with organic acid |  |  |  |  |  |  |  |  |
| Using fermented dry feed |  |  |  |  |  |  |  |  |
| Using fermented wet feed |  |  |  |  |  |  |  |  |
| Rough milled feed |  |  |  |  |  |  |  |  |
| Feed with high barley content |  |  |  |  |  |  |  |  |
| Home mixed feed |  |  |  |  |  |  |  |  |
| Acidified drinking water |  |  |  |  |  |  |  |  |
| High hygiene for workers, visitors, dogs, cats, tools |  |  |  |  |  |  |  |  |
| Rodent control |  |  |  |  |  |  |  |  |

*Question 5:*

*Which feeding system is your main system to your finishers? (Tick off one option)*

1. Home mixed wet feed restricted quantity
2. Wet feed based on purchased ready-mix restricted quantity
3. Home mixed dry feed with ad libitum quantity
4. Dry feed based on purchased ready-mix with ad libitum quantity
5. Other / multiple feeding systems

*Question 6:*

*Which flooring system do you have for your finishers?*

1. Solid floor in more than half of the area (and slatted in the remaining area)
2. Solid floor in less than half of the area
3. A combination of drained and slatted floor
4. Other / multiple flooring systems
